# Fighting for control in an unpredictable life – a qualitative study of older persons’ experiences of living with chronic dizziness

**DOI:** 10.1186/1471-2318-14-97

**Published:** 2014-08-29

**Authors:** Ulrika Olsson Möller, Eva Ekvall Hansson, Charlotte Ekdahl, Patrik Midlöv, Ulf Jakobsson, Jimmie Kristensson

**Affiliations:** 1 Center for Primary Health Care Research, Lund University, Jan Waldenströms gata 35, SE-205 02 Malmö, Sweden; 2 Department of Health Sciences, Health Sciences Centre, Swedish Institute for Health Sciences (Vårdalinstitutet), Box 157, SE-221 00 Lund, Sweden; 3 Department of Health Sciences, Lund University, Health Sciences Centre, Box 157, SE-221 00 Lund, Sweden; 4 Department of Clinical Sciences in Malmö, Lund University, Jan Waldenströms gata 35, SE-205 02 Malmö, Sweden

**Keywords:** Aged, Dizziness, Experiences, Support, Content analysis

## Abstract

**Background:**

Dizziness in older people is associated with disability and reduced quality of life. Few studies have investigated how daily life is affected from the older person’s perspective. Identifying barriers and resources in daily life could guide health care in how to direct efficient interventions. The aim of this study was to explore older persons’ experiences of living with chronic dizziness.

**Methods:**

In this qualitative study seven women aged 74–84 years and six men aged 73–87 years with chronic dizziness (≥3 months) recruited from a primary health care centre in 2012 participated in semi-structured interviews. The interviews were analysed by content analysis.

**Results:**

Interpretation of the interviews resulted in the overall theme “Fighting for control in an unpredictable life” with two themes. The first theme “Striving towards normality” revealed a struggle in daily life in searching for a cure or improvement and finding a way to maintain ordinary life. This process could result in feelings of resignation or adaption to daily life, and factors that supported living with chronic dizziness were described. The second theme “Having a precarious existence” revealed that daily life included being exposed to threats such as a fear of recurrent attacks or of falling, which resulted in an insecure and inflexible way of life. A feeling that symptoms were not taken seriously was described.

**Conclusions:**

The present study showed that older persons with chronic dizziness have needs that are not met by health care. Despite the fact that frequent contact with health care was described, the respondents described barriers in daily life that led to a restricted, inflexible and insecure daily life. Health care should therefore be individually tailored with focus on aspects of daily life, especially safety aspects. Support should also be continued until the older persons with chronic dizziness have developed coping strategies to gain control of their daily life.

## Background

Suffering from dizziness strongly affects the life situation as it is associated with disability and reduced quality of life, especially in older people [1-5]. Despite this, studies in older people aiming to explore the perception of the daily life situation from the perspective of those affected are sparse.

Dizziness is more common among older than younger persons. The prevalence varies across studies depending on study population and study design but a prevalence of 11–54% in older persons (aged 65 + years) has been reported, higher among women and increases with age [2,6,7]. Dizziness in older people are often persistent and difficult to treat [4,8,9]. In older persons the origin of dizziness may be difficult to establish and it sometimes has multiple causes [9,10]. Peripheral vestibular disorders with vertigo/dizziness as a primary symptom include benign paroxysmal positional vertigo (BPPV), vestibular neuritis and Ménière’s disease. Dizziness is also common in central neurological and cardiovascular disorders and psychiatric illness [11,12] and is a common side effect of medication [9]. A frequent and underdiagnosed cause of dizziness in older persons is multisensory dizziness [13]. Multisensory dizziness emerges due to the deterioration of multiple sensory receptor systems that occurs at old age and is established when all other vestibular disorders are excluded [4,10]. It involves a decline in postural control, which involves interactions of several systems, such as the musculoskeletal and sensory systems, with the purpose of stabilizing and orientating the body’s position in space. Dizziness in older persons is a predictor of disability [8,14] and falls [8,15,16]. It is also associated with functional aspects such as balance impairments [1], gait disturbances and problems getting outdoors [2] as well as need of help in personal and instrumental activities of daily living (PADL and IADL) [7]. Associated psychological factors were found to be fear of falling, depression and anxiety [1,2,7,8]. This shows that daily life is affected in many ways, physical as well as psychological, which may lead to limited possibilities to take part in different physical and social activities.

The complex aetiology of dizziness in older persons and the fact that dizziness may be a consequence of the aging process itself can lead to misunderstanding of dizziness in older persons as a natural and inevitable state. However, recent research has shown that it is not [7,17-20]. Dizziness in older persons has, due to its complexity, been described as a geriatric syndrome [4,9], a multifactorial health condition that occurs when the accumulated effects of impairments in multiple systems render a person vulnerable to situational changes [9]. When no single factor causing dizziness can be identified, treated and cured, a multifactorial strategy to identify contributing factors such as depression and balance and gait impairment, ought to be investigated with the aim of ameliorating the symptoms [1,4].

Few qualitative studies, especially in older persons, have explored daily life from the perspective of the individual. The experience of living with dizziness might differ between younger and older people because they are at different stages of life and thereby facing different challenges in daily life. Qualitative studies including both younger and older persons with peripheral vestibular disorders [21], vertigo [22] or disorders with central, peripheral or somatoform origins [23] showed that dizziness causes severe limitations in life [21]. They also showed that dizziness exerts effects on multifaceted aspects of functioning and disability [23]. In a study in people aged 65 + years with dizziness [24], stabilizing the symptoms and preserving mobility seemed to be important priorities. The same study revealed that the respondents were not well informed about possible causes of dizziness. However, that study mainly aimed at identifying expectations and wishes when consulting their general practitioner (GP) in primary health care and did not explore the daily life situation in older people with dizziness. A broader perspective might bring insights into a variety of aspects affecting daily life. This knowledge could provide a deeper understanding of the situation for older people with dizziness and the kind of support they are in need of, useful knowledge when designing health care for this group. The aim of the present study was to explore older person’s experiences of living with chronic dizziness.

## Methods

### Design

To gain more insights and increase the depth of understanding of how daily life is affected in older persons with chronic dizziness, a qualitative explorative design with interviews as the data collection method was used [25].

### Participants

The participants were purposefully chosen from a primary health care centre in a city in the south of Sweden. The purposeful selection in this context was chosen to recruit older persons with persistent dizziness that was difficult to treat, implying that daily life was affected. Efforts were made to include participants with variation in terms of gender, age, duration of dizziness and diagnosis. The inclusion criteria were: aged 65 + years, dizziness of any cause for at least three months, and ability to communicate in Swedish. Seven women aged 74–84 years and six men aged 73–87 years agreed to participate. They had suffered from dizziness for 1.5 and 20 years (median 7.5 years). Five had been diagnosed with Ménière’s disease, six with multisensory deficits, one with vestibular neuritis and one with acoustic neuroma by their GP or Ear, Nose and Throat (ENT) specialist. Most participants described multiple diseases including heart disease, polyneuropathy, asthma, cancer, arthritis, Guillain-Barré, diabetes, osteoporosis or depression. Five were cohabiting and eight lived alone. Four had home-help (cleaning) or help from relatives in instrumental activities of daily life (IADL). None of them had help in personal activities of daily living (PADL).

### Procedure

The recruitment procedure started when a physiotherapist made a follow-up call to persons that had received physiotherapy (PT) on account of dizziness. The respondents had been referred for PT by their GP, ENT specialist or by themselves. To minimize the risk that the respondents were in a state of dependency they were not in active PT treatment. At the follow-up call they were informed about the present study and asked whether they were willing to participate. The first author and interviewer (UOM) contacted them by phone, gave them information about the study and explained that they could withdraw at any time without giving any reason. During the phone calls two persons withdrew due to lack of time and one person due to not wanting to record the interview. For the remaining 13 participants an appointment was made for the interview and a letter with written information about the study, the interview and contact information was sent. The interviews were conducted between February and October 2012, 11 at the participants’ homes and two at the primary health care centre, according to the participants’ preferences. At the beginning of the study the first author and last author (JK) conducted one interview together. The last author took part as an observer to support the first author in terms of interview technique and to be able to discuss the interview guide in depth; all other interviews were conducted by the first author. The interviewers had no professional relationship with the participants. To focus on the aspects of living with dizziness a semi-structured interview guide (Appendix A) with some predefined questions and special topics [26] was developed and tested in two interviews in older people with dizziness (not included in this study) by the first author and was adapted and refined together with the last author. The guide was constructed with four themes: describing dizziness symptoms; the impact on daily life; strategies for managing daily life; and health care and support. The interviews started with the request: “Please tell me how the dizziness started.” and probing questions were added for clarification. The interviews were recorded and transcribed verbatim by the first author. The interviews lasted between 37 and 72 minutes (mean 54 minutes). To ensure confidentiality each respondent were labelled with a number, and the code list was kept in a secure file only available to the research group. Confidentiality was also taken in consideration when presenting the results.

### Data analysis

A qualitative content analysis was performed according to Graneheim and Lundman [27]. Content analysis may cover manifest or latent levels or a combination of both where the manifest content refers to the visible, obvious components and the latent component refers to an interpretation of the underlying meaning [26,27]. The analysis covered both manifest and latent levels and the components were labelled as subthemes, themes and overall theme [28]. The analysis was inductive and conducted in several steps (Table 1). All of the authors read the transcribed interviews. To become familiar with the interviews and get a deeper understanding as a whole, UOM listened to and read them several times. The content and general understanding of the text was confirmed by EEH and JK. The units of analysis were then identified and coded by UOM. To increase the credibility of the study, UOM and JK subthemed two interviews independently of each other and discussed the content until consensus was reached. The interviews were then labelled with preliminary themes and at this time all co-authors met and discussed the themes through back and forth movement between the whole and parts of the text [27] until consensus was reached. All authors participated in the development of the themes and the overall theme. To clarify the results, quotations from the interviews were used and were sometimes carefully edited. Ellipses parentheses (…) indicated removal of irrelevant words or short sentences and square brackets [] indicate clarifications by the authors.

**Table 1:** Example of the analytic process by which meaning units, codes, subthemes and themes were formed

**Meaning unit**	**Code**	**Subcategory**	**Category**
“This general feeling of dizziness, a little nauseous, I don’t know when I wake up what the day will be like (…) I don’t dare to do more than a couple of errands a day.”	Can only plan a few activities per day	To be restricted in everyday life	Having a precarious existence
“Oh, it would be lovely to listen to music or a lecture, because I want to keep up with things…but I cannot get out on my own.”	Cannot engage in social activities		
“There was follow-up, and what should we do now? I told them that the treatment had no effect, but I didn’t get…I have awaited their response but…maybe the treatment has ended, but then I want to know that it has…that there is nothing else they can do.”	The treatment had no effect and no further plans for the future were made	To get insufficient support	
“And I have to hold on to the banister when I walk up and down the stairs, but sometimes I have tried not to, because I think ‘now, damn it, away with it!’ but I just can’t…”	Trying to walk on stairs as before	A struggle to maintain or regain an ordinary life	Striving towards normality
“..I feel very frustrated that…my wife has to both take care of the garden here, and the garden is really quite large…and look after the house and the garden…car and all the things I used to do…”	Frustration when not managing things as before	Movement towards resignation or adaption	
“I have a bag with me which I hold in my right hand and then it’s easier for me to walk (…) and then I take longer steps (…) and when I have the bag, it swings in time with my body and then it feels better to walk.”	Walks better when holding a bag		

### Ethics

This study has been performed in accordance with the Declaration of Helsinki [29]. The study was approved by the Regional Ethics Review Board in Lund (approval nos. LU 2010/402 and nos. LU 2011/659). Written consent was obtained from all respondents.

## Results

**Figure 1:**
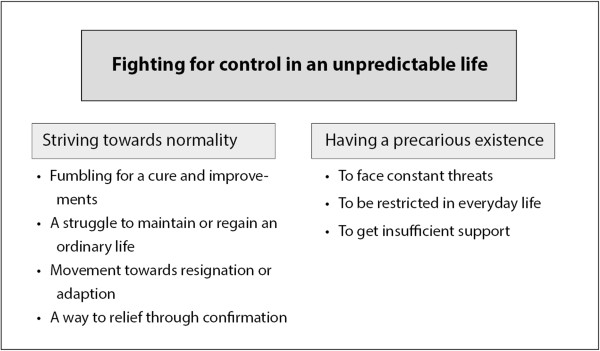
Thematic ovexrview of the results.

Living with chronic dizziness during old age was interpreted as *fighting for control in an unpredictable life*, which formed the overall theme. This theme constituted two themes; *striving towards normality* and *having a precarious existence*. These themes in turn comprised various subthemes (Figure 1): Thematic overview of the results.

### Striving towards normality

Living with dizziness was interpreted as fighting to live a normal life; that is to maintain or regain life the way it used to be before the dizziness started. It was expressed as recurrently trying out different strategies to cure, improve or control the dizziness symptoms. This could also mean holding on to previous activities or a specific and individual “way of life”. Inevitable changes in daily life were interpreted as voluntary or involuntary which strongly affected how living with dizziness was perceived. By getting support their concerns were taken seriously. This theme comprised four subthemes: *fumbling for a cure and improvements, a struggle to maintain or regain an ordinary life, movement towards resignation or adaption* and *a way to relief through confirmation*.

### Fumbling for a cure and improvements

Even if the respondents had suffered from dizziness for a long time and sometimes didn’t have acute recurrent attacks anymore, they still had a wish to be cured or improved and expressed a feeling of not being “well enough”. It was perceived as a disappointment to never become as well as they wanted to be. This was described as constantly trying out different ways to improve such as acupuncture and exercise but also using motion sickness bracelets, seeking new information about their symptoms or requesting a second opinion. A respondent who had suffered from dizziness for 20 years said:

R: “I feel like half a person and that’s why I have done so much to try and get rid of this feeling of dizziness, to feel normal in the head somehow…”

I: “That is your wish?”

R: “Yes, more than anything else, yes. That’s why I’ve tried so many things I really have…” (I3)

### A struggle to maintain or regain an ordinary life

The respondents sensed that they had to fight to live the life they wanted to live and not give in to the dizziness. One way of dealing with it was to fight to remain independent, which could mean not wanting to use aids or not accepting help from others. It could also mean trying out activities that had been manageable before, like climbing stairs without using the railing. Another way of dealing with this was to be determined and go through with planned activities as one responded noted:

R: “… we play boules once a week as I have done for 15 years and it’s hard to stop… later I should go home but THEN I’m tired, I’m exhausted, thinking ‘Will I make it home or won’t I make it home?’… It’s at the limit of what I can manage. So I just put my boules bag down – it’s a backpack – and take off my shoes and throw myself on the bed and then I am just totally exhausted.” (I5)

### Movement towards resignation or adaption

Some of the respondents expressed that suffering from dizziness negatively affected their life greatly. They felt resigned to the fact that they involuntarily were being forced to give up activities such as running a business, travel, gardening or social activities. It could also mean not being able to maintain their standard of living, as one respondent expressed:

R: “I can’t do anything I want, that’s the worst thing… I was not such a casual person that I would… I like things to be neat and tidy because I want things to be in order and so it’s so hard when you have dizziness, you can’t do what you want.” (I2)

Other respondents described that they voluntarily had adapted to life with dizziness. They sensed feelings of gratitude over the things they were still able to manage by themselves and of making the best of the current situation. Self-learned strategies such as taking it easy, avoiding stress, resting and physical exercise were used to adapt to life with dizziness. Other strategies included minimizing the risk of hazardous situations such as reducing the risk of falls by holding on to furniture, walking on an imagined straight line or looking at a fixed point. It was like learning a new way to move. Self-learned strategies to handle daily life with dizziness gave them control of the situation:

R: “I’ve developed a number of techniques so I can eliminate a lot of the effects [of dizziness].

I: “Mm.”

R: “So… I do not need… I am, because I am now aware of all these situations, I can in some way use those… techniques in that situation and I don’t need to be… so to speak… clueless, but I can instead say ‘Oh well, now I can imagine doing this and that’, or think this and that.” (I6)

### A way to relief through confirmation

Dizziness was sometimes described as an invisible handicap which sometimes made it difficult to receive help. Support was a confirmation of their perceived difficulties. Informal support was described as help with daily practical issues, children and grandchildren calling or helpful neighbors. It was also confirmed by receiving help from health care or community care, such as successful treatments, quick access to primary and specialist care, access to different aids, social services or home visits by health care providers. This was perceived as enhancing physical as well as psychological aspects. A respondent that had received PT said:

R: “It gives me a certain self-confidence when I go there and meet a nice person ‘Oh, you can manage, it’s OK’, ‘Yes, but I can’t do it’, ‘Yes you can, you have become much better’, it’s good to hear… (…)… you think… if I go there it will become better and better and by the end I’ll be… stronger… and won’t deteriorate eventually” (I7)

### Having a precarious existence

The second theme was interpreted as having an insecure daily life in terms of not knowing how the day or the future would turn out. This could mean a fear of getting into dangerous or unpleasant situations such as losing one’s balance when being outdoors, or concerns about being dependent on others in daily activities. It also involved a fear of not receiving the amount of help and support that was needed today or in the future. A lack of support from health care led to feelings of being abandoned and expectations of recovery being dashed. This theme comprised three subthemes: *to face constant threats, to be restricted in everyday life,* and *to get insufficient support*.

### To face constant threats

Facing constant threats was described as living with ever-present risks such as the sudden onset of recurrent vertigo attacks or a fear of falling with the risk of being injured or not being able to get up and call for help. A fear that a staggering walk might be thought of as drunkenness was mentioned. Future threats included not managing ADL, driving or living in one’s own house. Daily life involved worrying and sometimes dangerous situations, as one respondent described:

R: “I’m alone and for example can’t retrieve anything that’s high up and… I can’t bend down and sometimes I’m forced to kneel and then it’s hard to get up, and if I go out onto the street then I hope I won’t drop something because I can’t bend down…”

I: “But what do you do when you need something that’s high up? What do you do then?”

R: “Well… sometimes I use a stepladder, which I know I’m not supposed to…” (I7)

### To be restricted in everyday life

To live with dizziness included being forced to have a constant awareness of daily activities such as how to move or activities to avoid so as to not trigger dizziness. This led to everyday adjustments such as only planning one activity per day, planning periods of rest and having to adjust their activity level to the shape they were in that day. Having to make priorities in daily activities also meant excluding activities that they knew would be good for them and it was sometimes difficult to find the most appropriate activity level:

R: “Well, I have to cut back on some things, yes… that’s how I work, and it was the same… one day I’m weaving and it’s going really well, I’m sitting at the loom and I don’t feel anything, but when I get up it’s really hard, but I do it a whole afternoon and a bit more, and then I have aqua training, which I really try to attend. When I had [name of a physiotherapist] too it became too much for me… it simply became too much. It creates certain obstacles to exercise and so on” (I3)

Daily life could also be hampered by being dependent on others in daily activities such as cleaning or transport. Being dependent on others also caused feelings of frustration at being forced to adapt to others, or feeling obliged to lower ones demands so as to not burden relatives or friends. To live with constant need of aids for physical support, which sometimes meant having a security zone limited to an arm’s length, was described as a limitation. Other restrictions in daily life were inaccessibility of public buildings or social events, difficulties in using public transport, and having to avoid loud environments. Other environmental issues were darkness, crowds and uneven surfaces, which could exaggerate the feeling of dizziness. This led to the avoidance of activities, as one respondent expressed:

R: “So as soon as there is any unevenness… only going down into the gutter feels like, Oh… like an abyss, I can’t use the escalator, going up is OK, but not down, and not down stairs either because then I feel the abyss…”

I: “Mmm… then you’ve had to give up certain things, or…?”

R: “Yes, I’ve had to… like going to the theater where… at the concert hall there is no handrail to hold onto but… then you have to take a step to the next row of seats, it’s very bad for disabled people, both at the theater and elsewhere, at the cinema, and when it’s dark, it’s very difficult” (I4)

### To get insufficient support

Some of the respondents who had had dizziness symptoms for a long time described a lack of support from family, friends or neighbors. A lack of support from health care was also described, although frequent contacts with different health care organizations were reported. The respondents wanted further information about the cause and prognosis of the dizziness and also to be further investigated. They expressed that the dizziness symptoms were sometimes not taken seriously, which was described as a lack of competence, long waiting times and poor follow-up. They expressed that it was important to stay active, but to manage this by themselves was sometimes perceived as difficult and even hazardous. This was expressed as, for example a fear of walking outside alone or a fear of falling when doing home exercises after ending a PT session, as one respondent expressed:

R: “Yes, so when I sometimes feel… I stand there moving my head back and forth [home exercises]… then it hits me [the dizziness] and I have to be ready to grab the chair… or I will fall backwards into the wall” (I4)

Sometimes the treatments had no or only temporary effects. This was also expressed as not receiving the right kind or amount of treatment. Sometimes an improvement was seen in objective measures, for example after a PT session, but no improvement was experienced by the respondent, as one said:

R: “… she [physiotherapist] had a chart showing the changes during the time I went there, and then it were… according to it… the figures, it had got better…”

I: “Yes, it had”

R: “Yes, she had, basically, not all, but basically they had improved… whatever it came down to with these various… movements, but what I have… what she asked about and what I have… my current situation isn’t any better”

R: “No, OK”

R: “So that’s it, really… the chart and the measurements aren’t really very important, rather how I feel now”

I: “Exactly”

R: “That’s what’s important” (I13)

## Discussion

This qualitative study showed that living with chronic dizziness during old age could be interpreted as a fight to take control over one’s life situation when living an unpredictable existence. The fight was described in various ways by all respondents and how much daily life was affected varied. The unpredictability was, however, an aspect that they always had to consider and which strongly affected daily life.

The results of this study are in line with other studies giving a multifaceted picture of functioning and underlining how much the pathology of dizziness interferes with daily life [21,23,24]. Aspects of living with dizziness have been linked to over 140 International Classification of Functioning, Disability and Health (ICF) categories in bodily functions, activities and participation, as well as environmental factors [23] and ICF may be used as a way to describe the complex situation of the individual.

Striving towards normality was described as a variety of fighting strategies. It was expressed as trying to maintain or regain physical functions or great efforts to continue with certain activities, showing that they were fighting to maintain their previous lifestyle. Some of the respondents had to plan their activities in advance but this strategy was expressed as being very energy consuming and the respondents expressed concerns as to whether or not they would be able to go through with their plans. To stay independent was an important issue, as was shown previously [21]. Self-learned strategies were similarly described in other studies in persons with chronic dizziness [21,24], indicating that they are fighting on their own to take control of the situation. Earlier studies showed that dizziness is associated with decreased activities in daily living and decreased engagement in social activities [1,2,7]. The present study shows that, even after many years with dizziness, elderly sufferers are still fighting to regain the life they used to live before their dizziness started. This was seen in both “Fumbling for a cure and improvements” and “To get insufficient support”.

Fighting for control contained a physical dimension in terms of fighting to stay mobile or not fall, but also a psychological dimension in terms of “understanding their disease” or fighting to have their concerns taken seriously or to receive treatment. A wish to find the cause of dizziness was reported previously [21,24]. Even though the respondents in the present study had diagnoses not all of them knew the cause of their dizziness and this indicates the need to develop information strategies for older people with chronic dizziness.

The results reveal that the respondents needed continuous support. Even though the respondents had been diagnosed and medically treated, they still strived to be cured or improved. To identify and treat underlying potentially life-threatening conditions should always the main priority when examining and treating persons with dizziness [20,30], but the present study shows that the need for support remains even when there is no cure.

Supportive resources that counteracted the negative effects were described. They could include receiving support from family and friends, as was shown previously [23], but also support in relation to health care. Successful treatments and quick access to health care were, as expected, expressed as moderating factors. However, in addition, it was also important that one’s concerns were taken seriously, that there were somewhere to turn and that one was supported and encouraged to cope living with dizziness. This indicates that treatment should not end until the older person with dizziness has developed the ability and self-efficacy [31] to control their daily life situation.

Dizziness in older persons is associated with balance impairment, fear of falling, falls and fall-related injuries [2,8,32-35] and also forms of emotional vulnerability such as exhaustion and insecurity [21-23]. In the present study it clearly emerged that these are all aspects that may cause unpredictability in daily life. Internally the fear was manifested as a feeling of not being able to trust and control the body and externally it was manifested as environmental barriers, a fear of getting into dangerous situations or a lack of support. Due to those fears a majority of the respondents were not able to live life the way they wanted to and avoided making plans for the future, which made their daily lives strongly restricted, inflexible and insecure.

This article explored daily life in older people with chronic dizziness, but threats to control have been described in people suffering from other diagnoses such as Parkinson’s disease and diabetes [36,37]. However, managing threats in daily life in old age may require consideration of individual and contextual factors, and not merely the diagnosis [38]. Health care professionals should understand the significance of control and threats to control in an older persons’ life, and give individual support [38], that goes beyond merely treating a specific diagnosis. To focus on safety aspects of daily life could be one way to increase control.

Our results clearly indicate that health care has not managed to meet the respondents’ needs. Some of the respondents showed a strong will and motivation to fight to take control of their situation. Others felt resigned with no hope of recovery and lacked the ability and confidence to manage and control their situation. They all expressed difficulties in managing daily life. It is therefore important for health care personnel to pay attention to the state and specific requirements of the affected person to be able to tailor treatment. Like other studies [4,9], the present study emphasized that in chronic conditions interventions that influence daily life situations is more desirable than merely receiving a diagnosis-specific treatment. Elderly people with dizziness need support to be able to solve everyday situations and to adapt to a new life situation. For some respondents this could include solving practical issues or support to stay active and independent. Others who expressed feelings of resignation and not being in control of their situation might be in need of other treatment methods. Combining physical and psychological therapies in older patients with dizziness has shown positive results [39].

### Methodological considerations

The qualitative design of the present study implies that trustworthiness in term of credibility, transferability, confirmability and dependability has to be taken in consideration [27,40]. Credibility refers to the truth and believability of the data and was enhanced through variation in gender, age and cause and duration of dizziness. Even though the age variation might be considered quite small, the variation in duration of dizziness and cause of dizziness increased credibility. The study included 13 respondents and sampling was continued until saturation, i.e. no further information was obtained. Credibility was enhanced by triangulation i.e. the researchers involved in this study had different professional backgrounds, all with experience of working with older persons in different health care settings and some with further education in vestibular disorders. Credibility was also enhanced by the transparent description of the analytic process (Table 1). To ensure dependability i.e. the stability of data, a thematic interview guide (Appendix A) covering different topics with the same open initial question was used to ensure that the interviews covered the same areas. Confirmability refers to the objectivity of the researcher and the data analysis and was enhanced through researcher triangulation and the use of quotations from the respondents’ statements.

### Clinical implications

This study revealed that when managing older people with chronic dizziness in clinical practice it is important to pay attention to the state and specific requirements of the individual to optimize the care. Barriers and resources in daily life must be taken in to consideration. Treatment should not end until the person has developed the ability to control the daily life situation. The care should specifically focus on enhancing safety aspects in daily life. This study also highlights the need to improve how information is communicated to older people with dizziness.

## Conclusions

This study showed that even though the respondents in this study had been diagnosed and medically treated, they still strived for a cure or improvements. This implies that health care for older people with dizziness do not cover their needs. To maintain their previous lifestyle and stay independent was considered important. Enhancing safety and control in daily life should be prioritized. The support for older persons with chronic dizziness should therefore be individually tailored and not end until they have developed coping strategies to control their daily life.

## Appendix A

Semi-structured interview guide

Theme 1: Describing dizziness symptoms

Please tell me how the dizziness started.

Please tell me how it is living with dizziness today.

Can you describe your dizziness without using the word dizziness?

Theme 2: The impact on daily life

How is everyday life affected by your dizziness symptoms?

How was your life situation before and dizziness started? And now?

What would your life be like today without dizziness?

Theme 3: Strategies for managing daily life

Please tell me about a dizziness episode. What happened? What did you do then?

Theme 4: Health care and support

Please tell me about your contact with health care due to dizziness

Do you think your future life will be affected by the dizziness? How?

## Competing interests

The authors declare that they have no competing interests.

## Authors’ contributions

UOM was responsible for the study design, acquisition and analysis of data and drafting the manuscript. JK substantially contributed to the study design, acquisition and analysis of data and drafting the manuscript. EEH contributed to the study design, interpretation of data and in drafting the manuscript. CE, PM and UJ contributed to the study design and drafting the manuscript. All authors critically revised the manuscript and approved the final version to be published.

## Pre-publication history

The pre-publication history for this paper can be accessed here:

http://www.biomedcentral.com/1471-2318/14/97/prepub
